# Sleep and Mental Disorders

**DOI:** 10.1192/j.eurpsy.2022.41

**Published:** 2022-09-01

**Authors:** T. Pollmächer

**Affiliations:** Klinikum Ingolstadt, Center of Mental Health, Ingolstadt, Germany

## Abstract

Since ancient times, the close connection between disturbed sleep and mental disorders has been well known. And yet, it was not until the middle of the 20th century that these connections could be studied in detail, after the EEG and REM sleep were discovered. Especially the study of the sleep EEG of depressed patients has brought to light alterations of SWS and REM sleep that could be of considerable clinical value, but at least in part still await broad confirmation. In the treatment of disturbed sleep in psychiatric patients, cognitive behavioral therapy in particular has become increasingly established in recent years, whereas only limited innovations have been recorded with regard to pharmacological treatment. Only in recent years it has become clear that psychiatric disorders are often accompanied by clinically relevant somatic disorders of sleep, such as obstructive sleep apnea syndrome, and that such comorbidities urgently require attention and treatment. It is becoming increasingly clear that detailed knowledge of sleep disorders is essential for the effective treatment of mental disorders. Moreover, it is likely that the scientific study of sleep in mentally ill people still has untapped potential in terms of understanding the pathophysiology.

**Disclosure:**

No significant relationships.

